# Sparse tensor phase space Galerkin approximation for radiative transport

**DOI:** 10.1186/2193-1801-3-230

**Published:** 2014-05-07

**Authors:** Konstantin Grella

**Affiliations:** Seminar for Applied Mathematics, ETH Zurich, CH-8092 Zurich, Switzerland

**Keywords:** Radiative transfer, Sparse grids, Discrete ordinates method, Spherical harmonics method, Combination technique

## Abstract

**Abstract:**

We develop, analyze, and test a sparse tensor product phase space Galerkin discretization framework for the stationary monochromatic radiative transfer problem with scattering. The mathematical model describes the transport of radiation on a phase space of the Cartesian product of a typically three-dimensional physical domain and two-dimensional angular domain. Known solution methods such as the discrete ordinates method and a spherical harmonics method are derived from the presented Galerkin framework. We construct sparse versions of these well-established methods from the framework and prove that these sparse tensor discretizations break the “curse of dimensionality”: essentially (up to logarithmic factors in the total number of degrees of freedom) the solution complexity increases only as in a problem posed in the physical domain alone, while asymptotic convergence orders in terms of the discretization parameters remain essentially equal to those of a full tensor phase space Galerkin discretization. Algorithmically we compute the sparse tensor approximations by the combination technique. In numerical experiments on 2+1 and 3+2 dimensional phase spaces we demonstrate that the advantages of sparse tensorization can be leveraged in applications.

**2010 Mathematics subject classification:**

35Q79; 65N12; 65N30; 65N35

## Introduction

In this paper, we consider the numerical solution of the radiative transfer problem (RTP). This transport problem is stated on the phase space  as the Cartesian product of a bounded physical domain , where *d*=2,3, and the unit -sphere as the parameter domain  of dimension . The RTP (see e.g. Modest 2003) is then given as the task of finding the unknown *radiative intensity*, a real function over the phase space satisfying

1a1b

We refer to Eq. (1a) as the *stationary monochromatic radiative transfer equation* (RTE), while Eq. (1b) constitutes *inflow boundary conditions*. A ray of light of direction ***s*** is attenuated by absorption and scattering with the medium. In (1a), *κ*≥0 is the *absorption coefficient*, *σ*≥0 the *scattering coefficient*, and *Φ*>0 the *scattering kernel* or *scattering phase function*. The scattering phase function is normalized to  for each direction ***s***. Sources inside the domain *D* are modeled by the *blackbody intensityI*_*b*_≥0, radiation from sources outside of the domain or from its enclosings is prescribed by the *boundary datag*≥0. The vector ***n***(***x***) denotes the *outward unit normal* vector which is defined in (almost every) point ***x*** on the boundary *∂D* of the physical domain.

Due to the high dimension of the phase space, the nonlocality of the scattering operator, and the hyperbolic nature of the PDE, the efficient numerical simulation of radiative transfer is a challenging computational task even today. Still, radiative transfer as such or as a means of energy transfer among others is of interest in many applications, e.g. in the fields of heat transfer (Modest 2003), neutron transport (Hébert 2010), atmospheric sciences (Evans 1998), medical imaging (e.g. Peng et al. 2011), or other areas where transported particles interact with a background medium, but only negligibly with each other.

In this paper, we extend the range of sparse tensor product discretization methods for the RTP investigated before (Grella and Schwab 2011a; Grella and Schwab 2011b; Grella 2013; Widmer et al. 2008) by a new phase space Galerkin framework.

Apart from Monte Carlo methods for raytracing, the most popular deterministic approaches for the radiative transfer problem are the discrete ordinates method and the spherical harmonics approximation. We quote a brief overview of their properties from (Grella 2013).

In the discrete ordinate method (DOM) or *S*_*N*_-approximation, the angular domain is discretized by a number of fixed directions, which are inserted into Eq. (1) so that a system of spatial PDEs results. Without scattering the equations for single directions are independent of each other, with scattering, however, they are coupled through the scattering integral. After the straightforward discretization of the angular domain, the spatial PDEs are typically solved using finite differences, finite elements, or finite volume methods.

The DOM is popular as it is simple to implement, offers straightforward parallelization, and can capture directed radiation relatively well as some of the ordinates can usually be chosen freely.

On the downside, the method can suffer from so-called “ray effects” (Lathrop 1968): due to the point evaluation in the angular domain, the scalar flux or incident radiation from small isotropic sources may appear star-like with rays emanating from the source into the chosen angular directions (Stone 2007, p. 2 and following). These effects occur especially pronounced in settings with low scattering and absorption, i.e. in optically thin media.

An example for truncated series expansion is the method of spherical harmonics or *P*_*N*_-approximation. The solution of Eq. (1) is replaced by a series of spherical harmonics up to some order *N* with spatially dependent coefficients. Due to orthogonality relations, the scattering part often decouples or couples only few terms depending on the scattering kernel. However, the system of PDEs for the spatial coefficient functions is always coupled by the transport part ***s***·***∇***_*x*_*u*.

As low order series expansions in spherical harmonics do not permit a very localized resolution of the angular variable, the method performs best when the solutions are nearly isotropic in angle, which is the case in diffusive, so-called “optically thick” media. Then, rather low order spherical harmonics approximations might suffice for a good approximation. Indeed, the *P*_1_ method can be formulated as a diffusion equation for the incident radiation (Modest 2003, Sec. 15.4). For smooth solutions, the spherical harmonics method exhibits spectral convergence in angle (Grella and Schwab 2011a).

On the other hand, beam-like solutions require a high spectral order to be resolved appropriately, leading to high computational complexity. In general, higher spectral orders also lead to a sharp increase in computational complexity when boundary conditions are to be satisfied (Modest and Yang 2008).

When combined with a standard finite element or finite volume discretization in the physical domain *D*, the deterministic, numerical *S*_*N*_- and *P*_*N*_-approximations exhibit the so-called “curse of dimensionality”: the error (typically the *L*^2^-error of the solution) with respect to the numbers of degrees of freedom (DoF) *M*_*D*_ and  on the component domains *D* and  scales with the dimension *d* and  of the application problem as  with constants *s* and *t*.

The first sparse finite element approximation method was proposed in (Zenger 1991) for the solution of Laplace equation in the unit square and cube. In this paper, Zenger developed the (direct) sparse grid approximation method which alleviates this curse of dimensionality: the computational complexity is reduced, up to logarithmic terms, to that of a one-dimensional problem.

The idea of sparse tensorization of finite element and finite difference methods was generalized by (Bungartz and Griebel 2004; Hegland 2003; Garcke 2007), and others, for the numerical solution of PDEs as well as for other applications where standard numerical methods are obstructed by the curse of dimensionality.

Sparse tensor methods were first applied to radiative transfer by (Widmer et al. 2008). In that paper, the authors formulated a least squares phase space Galerkin sparse tensor approximation with hierarchical finite elements as discretization of the physical domain and wavelets in the angular domain. They proved that sufficient regularity of the solution provided, their method breaks the curse of dimensionality: the problem complexity reduces to log-linear in the number of degrees of freedom, while convergence rates deteriorate only by a logarithmic factor. However, the discretization of the scattering operator had not been addressed in that work.

In earlier work (Grella and Schwab 2011a), we showed that the sparse tensor product method of (Widmer et al. 2008) can also be combined with a spectral discretization involving spherical harmonics, resulting in a sparse *P*_*N*_-method which also treats scattering. Boundary conditions were satisfied in a strong sense by introducing piecewise spectral functions in angle.

Secondly we presented a sparse tensor version of the DOM as a sparse collocation method with a Galerkin ansatz in the physical domain and strong enforcement of the boundary conditions, while not yet accounting for scattering (Grella and Schwab 2011b). This sparse tensor *S*_*N*_-method was realized computationally with the sparse grid combination technique (Griebel et al. 1992) to construct a sparse approximation to the radiative transfer solution.

The sparse DOM was subsequently reformulated as a phase space Galerkin method with quadrature in angle (Grella 2013) in order to treat sparse *P*_*N*_- and sparse *S*_*N*_-method in a more uniform manner. In this reformulation, we employed streamline upwind Petrov Galerkin (SUPG) stabilization and weak satisfacion of boundary conditions. Sparse *S*_*N*_-methods were derived as a direct sparse tensor method and implemented algorithmically via the combination technique.

In the present paper, we derive a sparse *P*_*N*_- and sparse *S*_*N*_-method from the same phase space Galerkin framework with transport stabilization and scattering. Boundary conditions are satisfied in a weak sense. In doing so we close a gap in the list of conceivable combinations of formulations regarding stabilization and type of angular approximation. In contrast to the previous approach (Grella 2013), we stabilize the formulation in a different way and the analytical focus will be on the direct sparse approach. With transport stabilization and direct sparse approach we follow (Widmer et al. 2008) more closely, extending their work by treatment of scattering and weak satisfaction of the boundary conditions.

Similar savings in computational effort are realized with other variational formulations, such as Petrov-Galerkin saddle point formulations (see e.g. Dahmen et al. (2012) and the references there).

The outline of this paper is as follows. In Section ‘Phase space Galerkin method’ we formulate the phase space Galerkin framework in operator form and outline how *P*_*N*_ and *S*_*N*_-methods can be derived from it. Then we develop full tensor and sparse tensor discretizations based on the framework and analyze and compare their convergence properties.

Section ‘Numerical experiments’ presents several basic numerical experiments designed with the purpose of validating and illustrating the theoretical convergence results.

Finally we conclude this work in Section ‘Conclusion’ by summarizing and reviewing the results.

## Phase space Galerkin method

We begin by introducing the radiative transfer problem in operator form. Using this compact notation we then state the variational formulation of our phase space Galerkin method and proceed to discretizations of the method.

### Operator formulation

Problem (1) reads in operator form: Find the intensity  such that 2

In this, *∂Ω*_−_ represents the inflow part of the boundary  of the computational domain or *phase space*. The *inflow boundary* is defined by 3

with the *physical part of the inflow boundary*4

Correspondingly we define the *physical part of the outflow boundary* as 5

The *radiative transfer operator* A=T+Q consists of the *transport operator* T, 6

and the *scattering operator* Q, 7

Here, *Q*_1_=Id−*Σ* is the *unity scattering operator*, and *Σ* is the *scattering integral operator*, the integral of *Φ* and *u*. The *source functionf* contains the sources of radiation in the domain, 8

and *g* is the *incoming radiation* on the boundary *∂Ω*_−_, as in Sec. ‘Introduction’.

### Properties of the scattering operator

Aside from the positivity and normalization requirements already mentioned in Sec. ‘Introduction’, we assume an isotropic medium, i.e. *Φ* does not depend on ***x***. As *Φ* models the type of scattering, this assumption can safely be made for most applications (cf. Modest 2003, p. 268). Variations in the strength of scattering due to e.g. varying spatial density of the medium are modeled by the scattering coefficient *σ*. As long as the following properties hold for almost every ***x***, the complexity and convergence analysis later on could also be conducted without this assumption.

Furthermore, if spherical scatterers are assumed, the scattering phase function does not vary with the azimuthal angle so that *Φ* only depends on the inner product of ***s*** and ***s***^′^. From this it follows immediately that *Φ*(***s***,***s***^′^)=*Φ*(***s***·***s***^′^)=*Φ*(***s***^′^,***s***).

From here on, we shall take *Φ* (cf. Kanschat to be *forward dominant*^2008^, Def. 1) if  with all *a*_*k*_≥0. Then, one can show that *Σ* is positive semi-definite (Kanschat 2008, Lemmata 2 and 3), i.e. 9

Normalization and symmetry of *Φ* with respect to its arguments leads to normalization of the operator norm  (Kanschat 2008, Lemma 5).

From these properties and a Hilbert-Schmidt theorem for integral operators (e. g. Knapp 2005, Thm. 2.4), one can derive that the spectrum of *Q*_1_ lies in [ 0,1] with an isolated eigenvalue *λ*_0_=0, from which the next largest eigenvalue *λ*_1_ differs by a positive constant (Ávila et al. 2011, Sec. 2.2).

With the previous considerations, one arrives at the following properties of Q:

**Lemma 1.***For anyu*∈*L*^2^(*Ω*), *the scattering operator Q as defined by Eq.* (*7*) *satisfies (cf. Ávila et al.*^2011^, *Eq*. (11*))*1011

in which the projector *P*^⊥^ maps  to (kerQ)^⊥^, the space orthogonal to the kernel of Q, and *λ*_1_∈(0,1] is the smallest nonzero eigenvalue of *Q*_1_.

For a proof of (11) we refer to (Grella 2013).

### Variational formulation

Our variational formulation will be based on a Galerkin finite element framework over the phase space *Ω* with stabilization applied to the operator RTP (2).

#### A generic stabilized phase space variational formulation

To begin with, we define the Hilbert space 12

with the usual *L*^2^(*Ω*) inner product 13

and the *triple bar norm*14

For the weak enforcement of boundary conditions, we define the boundary form 15

in which we have omitted the dependence of the outward unit normal ***n*** on the position ***x***. This boundary form was introduced by Manteuffel et al. (2000, Eq. (2.16)). It is well-defined for functions *v*∈*L*^2^(*Ω*) with finite *inflow norm*16

Combining (14) and (16) yields the new norm 17

which gives rise to the closed, linear subspace of , 18

which, with the inner product related to ∥*v*∥_1_, is a Hilbert space. For functions , we define the bilinear form 19

where R is a stabilization operator on the test function side yet to be specified. Together with the linear form 20

the bilinear form constitutes the following variational problem: Find  such that 21

Different ways of stabilization are conceivable and have been used in the literature, e. g. the least squares approach by (Manteuffel et al. 2000), or SUPG introduced by (Brooks and Hughes 1982). For our purposes here, we will choose the T-stabilized formulation (Grella and Schwab 2011a) to avoid mesh-dependent quantities and the square of the scattering operator. More precisely, we set R=*ε*T with a stabilization parameter *ε* that depends on the absorption coefficient *κ*.

#### Properties of the variational formulation

At this point, we introduce the *anisotropic* or *mixed Sobolev spaces* as 22

with the corresponding *mixed Sobolev norms*, given by 23

Here,  denotes the weak derivative of  of order |***α***| w. r. t. ***x***∈*D* and order |***β***| w. r. t. , with the multi-indices  and .

The following lemma collects auxiliary results which will become helpful later.

##### **Lemma 2** (Auxiliary results).

1. Let . Then . If furthermore , then .

2. For *v*∈*H*^1,0^(*Ω*), it holds .

*Proof*. 1. A proof is given by (Manteuffel et al. 2000, Thm. 2.1). It uses the divergence theorem and exploits the fact that  for ***s***·***n***(***x***)<0 if , where ***n***(***x***) is the outward unit normal on the boundary *∂D*: 

As ***s***·***n***≥0 in the second integral, we obtain the first assertion. If additionally , then the first integral vanishes, and the second assertion follows.

2. We again quote Manteuffel et al. (2000, Lemma 4.1 (i)):  □

In order to establish well-posedness of the variational formulation (21), we prove continuity and coercivity of the bilinear form (19) and continuity of the linear form (20) in the following.

**Lemma 3** (Continuity of bilinear form). Let *σ*, *κ*, *ε*∈*L*^*∞*^(*D*) with , , , then there is a constant 0<*c*_*c*_<*∞* such that for all 

*Proof*. We proceed analogously to (Manteuffel et al. 2000, Thm. 3.3). To begin with, we estimate for all 2425

Using the Cauchy-Schwarz inequality as well as estimates (24) and (25) it holds  □

**Lemma 4** (Continuity of linear form). *Given the assumptions of Lemma 3 onκ*, *σ*, *ε*, *and additionallyf*∈*L*^2^(*Ω*), *with* ∥*g*∥_−_<*∞*, *there is a constant* 0<*c*_*l*_<*∞such that for**it holds*

*Proof*. The proof is analogous to that of Lemma 3:  □

Next, we show coercivity of the bilinear form. For ease of exposition we shall assume *ε* and *κ* to be constant on the physical domain. Coercivity can also be obtained for non-constant coefficients (see Widmer 2009, Thm. 2.2, for an example). Coercivity of the SUPG variational formulation for the RTP has also been proved by (Ávila et al. 2011, Lemma 2), although in a different norm. Previously, we had proved coercivity of the T-stabilized variational formulation without the boundary form *b*(·,·) (Grella 2013, Lemma 4.1), here we include this boundary form in the formulation, which will motivate the choice of the stabilization parameter *ε*.

**Lemma 5** (Coercivity of bilinear form). *Letκ*, *εbe positive functions which are constant on the physical domainD*. *Assume min****x***∈*Dσ*=:*σ*_min_>0 *and*, *and additionally that*26

*Then the bilinear forma*(·,·) *from (19) is coercive on*: *there is a constantc*_*e*_>0 *such that for all**it holds*

*Proof*. For an overview of the involved terms we split the bilinear form into separate inner products: 27

As we assumed *ε* and *κ* to be constant, we can factor these coefficients out of the inner products. We begin by analyzing the sum of first and fifth inner product.

Applying statement 1 of Lemma 2 yields

Together with the boundary term, we obtain 

The second inner product is bounded from below by 

To estimate the third inner product, we use property (11) of the scattering operator: 

The fourth inner product in Eq. (2,7) is 

For the sixth inner product we apply Cauchy-Schwarz inequality and Young’s inequality with a parameter *θ*>0: 

Combining all estimates yields the result: 

By eliminating *θ* we obtain the condition . The condition *ε*<2/*κ* results from the last of the terms over which the minimum is taken.

The previous condition on the stabilization parameter leads to a choice of *ε*=1/*κ*. Well-posedness of the variational formulation now follows directly.

**Theorem 6** (Existence and uniqueness of solution to variational formulation). *Provided thatf*∈*L*^2^(*Ω*) *and* ∥*g*∥_−_<*∞there exists a unique solution**to the variational formulation (21).*

*Proof*. Since  is a Hilbert space and Lemmata 3–5 guarantee continuity of the augmented SUPG bilinear form and linear form as well as coercivity of the bilinear form, the Lax-Milgram theorem (Brenner and Scott 2008, Thm. 2.7.7) ensures existence and uniqueness of the solution to (21). □

### Discretization

For the discretization of the variational problem (21), we restrict the space  in the variational formulation (21) to tensor products of hierarchic, finite dimensional approximation spaces over the component domains *D* and .

#### Full tensor discretization

In the standard full tensor approximation, we choose a full tensor product space *V*^*L*,*N*^ to approximate : 28

As  is a dense subspace of , we define the family of physical approximation spaces as 29

the spaces of continuous, piecewise linear functions on a dyadically refined mesh  over *D*. Here, the parameter *l*_*D*_ stands for the physical resolution. It is related to the mesh width *h* in  by . With respect to the resolution *l*_*D*_=0,…,*L*, the spaces  form a nested sequence 

Let  denote the number of degrees of freedom for the FE space  in the physical domain *D*. Then 30

with the dimension *d* of the physical domain. The exact number will depend on the geometry of the domain. For a square or cube *D*=[0,1]^*d*^, respectively, we obtain 31

In the angular domain, we distinguish between the *P*_*N*_-method and the *S*_*N*_-method.

##### ***S***_***N***_-method

Here, the family of approximation spaces is given by 32

the spaces of piecewise constant functions on a dyadically refined mesh . As the physical spaces, these spaces exhibit a nested structure. The angular resolution *N* and the dimension of  are related by 33

***P***_***N***_**-method** To define the angular approximation spaces of the *P*_*N*_-method, we first introduce the spaces of spectral functions of the -sphere, 34

where  are the spherical harmonics of the -sphere, and  is the largest value of the secondary index *m* depending on the value of the primary index *n* and the dimension. These spaces offer an inherent nested structure. To obtain the same relation (33) between resolution level and degrees of freedom as in the *S*_*N*_-method, we connect the resolution level *N* and  by .

Then, the angular approximation spaces are 35

and relation (33) also holds here. Up to the index relabeling and the additional boundary form, we obtain the spherical harmonics method already analyzed by (Grella and Schwab 2011a).

In both methods, the full tensor approximation space consequently has the dimension 36

The full tensor approximate solution can be expressed by means of a physical basis  of  and an angular basis  of  as 37

with solution coefficients . The *discrete variational formulation* finally reads: Find *u*_*L*,*N*_∈*V*^*L*,*N*^ such that 38

with the bilinear form *a*(·,·) from (19) and the linear form *l*(·) from (20). As *V*^*L*,*N*^ is a subspace of  well-posedness ensured by Thm. 6 for the continuous problem follows also for this discrete problem.

By choosing a subset of  as trial space we effectively assume a slightly higher regularity on the solution than what is guaranteed by the definition (12) of . For instance, solutions with line discontinuities due to the transport of discontinuous boundary data into the domain are not included in *V*^*L*,*N*^. However, since *V*^*L*,*N*^ is dense in , even discontinuous solutions will be approximated with increasing resolution. Furthermore, in order to leverage the advantages of a sparse tensor approximation, a higher regularity of the solution will be required in any case.

#### Equivalence of collocation DOM and phase space Galerkin DOM with quadrature

Ordinarily the discrete ordinates method is presented as a collocation method in angle: Fixed directions , , are inserted into the RTE (1a), and for each direction, the intensity  is sought as the solution to a purely spatial PDE. In these PDEs, the scattering integral is replaced by a quadrature rule 39

with weights *w*_*m*_>0. By applying a Galerkin ansatz with stabilization in the physical domain to the PDEs, a system of coupled variational formulations 40

results with directional stabilization and transport operators 41

In the phase space Galerkin approach, variational formulation (38) is discretized further by substituting the angular quadrature rule (39) for all angular integrals so that the bilinear form (19) is approximated by 

Let the linear functional *l*(·) from (20) be approximated by a functional  with angular quadrature correspondingly, then the directional solutions *u*_*j*_ are determined from the variational formulation with angular quadrature 42

Since this formulation has to hold for all *v*∈*V*^*L*,*N*^, it follows that for test functions which vanish at every angular quadrature node ***s***_*i*_,  except one *s*_*j*_, formulation (42) can be reduced to the variational formulation (40) from the collocation discretization. This condition on the test functions is satisfied e. g. for a basis of the test space of characteristic functions on the angular mesh if each mesh cell contains exactly one angular quadrature node. With such a one-point quadrature rule and characteristic basis functions of , the phase space Galerkin DOM is therefore equivalent to the collocation DOM after discretization.

#### Sparse tensor discretization

The full tensor approach presented before shows the typical complexity for full tensor approximations: The number of degrees of freedoms increases exponentially with the dimension and the resolution levels in a dyadically refined scheme.

A way to counter this exponential increase is found in sparse tensorization. Using the same approximation spaces on the component domains  and  as for the full tensor approximation we define a sparse tensor approximation space  by 43

where the *sparsity profile* determines which tensor product subspaces  are to be included in the approximation. The sparsity profile usually depends on *N* as well. Here, we employ a linear profile 44

which is normally chosen if the component complexities *M*_*D*_ and  depend on the resolution parameters *L* and *N* in the same way and identical order of approximation is sought over both component domains (cf. Zenger 1991; Bungartz and Griebel 2004; Griebel and Harbrecht 2013a).

If direct sum decompositions of the component approximation spaces  and  into detail spaces  and , i. e. 

 are available (correspondingly in the angular domain), then the sparse tensor approximation space  can also be written as 45

By choosing hierarchical bases for  and , each degree of freedom *u*_*ij*_ can directly be associated with a tensor product detail space . The sparse solution is then given by 

Thus, the *sparse discrete variational problem* reads: Find  such that 46

The dimension of the sparse tensor product space  depends on the sparsity profile . For a linear sparsity profile as in (44), the following complexity estimate is known (e. g. Bungartz and Griebel 2004, Lemma 3.6), or Griebel and Harbrecht (2013a, Thm. 4.1)).

**Lemma 7.***Assuming the dimensions of the detail spaces**and**scale as**with constantsc*_*i*_>0 *and dimensionsd*_*i*_, , *withd*_*D*_=*d*, *and given a linear sparsity profile**as in (44), the dimension of the sparse tensor product approximation space**as defined by (45) is*47

*whereθ*=1 if *andθ*=0 *otherwise. Relation “ ≲” defines an order up to constants with respect to the relevant scaling parametersL*, *N*: *a*≲*b* iff *a*≤*Cbwith constantCindependent ofL* and *N*.

### Error analysis

In this section, we shall show that the convergence rates of the full tensor and sparse tensor Galerkin methods differ only by a logarithmic factor in the degrees of freedom, provided that somewhat stronger regularity requirements are met for the exact solution.

The analysis will proceed along the usual fashion, cp. (Bungartz and Griebel 2004). We define the *Galerkin projector* into the full tensor product approximation space 48

Letting *L*→*∞* (*N*→*∞*) the fact that the subspaces are closed and dense implies that in the respective limits we obtain *semidiscrete Galerkin projectors* on the physical (angular) domain, as the Galerkin projector is stable in the ∥·∥_1_-norm:

**Lemma 8** (Stability of the Galerkin projector). *Let*. *Then there is a constantc*_*P*_>0 *independent ofLandNso that*

*Proof*. With continuity (Lemma 3) of the bilinear form we obtain 

Since this holds for all *v*_*L*,*N*_∈*V*^*L*,*N*^, we can set *v*_*L*,*N*_=P^*L*,*N*^*v* and exploit coercivity of the bilinear form (Lemma 5): 

If P^*L*,*N*^*v*≠0 we obtain the result with *c*_*P*_=*c*_*c*_/*c*_*e*_. □

#### Error estimates on the physical domain

To begin with, we require some approximation results in the *H*^1^(*D*)-norm on the physical domain. With a Clément-type quasi-interpolation operator  (Scott and Zhang, 1990, Thm. 4.1 and Cor. 4.1) we obtain

**Lemma 9** (Approximation of quasi-interpolation). *For polyhedral**and a shape-regular triangulation**onDwith mesh widthh*=2^−*L*^, *the quasi-interpolation**of a functionv*∈*H*^*s*+1^(*D*), *s*∈ [ 0,1], *to the space**of piecewise affine functions on**satisfies the error estimate*

 where *c*_*H*_>0 is a constant independent of *L*.

**Lemma 10** (Stability of quasi-interpolation). *Under the assumptions of Lemma 9, quasi-interpolation isH*^1^*-stable, i.e. there exists a constantc*_*B*_>0 *independent ofLsuch that for allv*∈*H*^1^(*D*) *it holds*

Next we derive an error estimate for the Galerkin approximation on the physical domain. At this point, the approximation is semidiscrete.

**Lemma 11** (Error estimate for Galerkin projection on physical domain). *Letu*∈*H*^*s*+1,0^(*Ω*), *s*∈{0,1}, be the exact solution to problem (21) and *the Galerkin projected solution to*49

with *a*(·,·) *from (19) andl*(·) *from (20). Then, there is a constantc*_*p*_>0 *independent ofLsuch that*

*Proof*. The proof is standard, and is based on coercivity and Galerkin orthogonality. We proceed analogous to (Ávila et al. 2011, Lemma 3 and Theorem 1). After inserting the quasi-interpolated solution  with  from Lemma 9 the triangle inequality permits us to write 50

For the first part, we use the fact that there is a constant *c*_*n*_>0 for all  such that 

Thus, we can apply Lemma 9: 

For the second part in (50), we use coercivity of the bilinear form, then in a second step Galerkin orthogonality, and finally continuity of the bilinear form to write 

and therefore with Lemma 9

By inserting into (50) we arrive at the result  □

#### Error estimates on the angular domain

On the angular domain, the considerations in the following require an approximation result for *L*^2^-projections.

**Lemma 12.***For functions*, *t*∈{0,1}, *theL*^2^*-projection to the space**satisfies the error estimate*51

where the constant *c*_*l*_>0 is independent of *N*.

This result can be obtained for approximation by spectral functions as in the spherical harmonics method (in which case *t*≥0 is arbitrary), for instance, as well as for approximation by piecewise constants as in the discrete ordinates method (in which case 0≤*t*≤1). It allows the derivation of the same approximation rate for the semidiscrete Galerkin projection on the angular domain.

**Lemma 13** (Error estimate for angular Galerkin projection). *Letu*∈*H*^1,*t*^(*Ω*), *t*∈{0,1}, *be the exact solution to problem (21) and**the Galerkin projected solution with angular part from the subspace**of*. *Then there is a constantc*_*a*_>0 *independent ofNsuch that*

*Proof*. The proof proceeds analogously to the one of Lemma 11 while substituting the *L*^2^-projected solution with Lemma 12 for the quasi-interpolated solution, the details are therefore omitted here. □

#### Error estimate for the full tensor phase space Galerkin method

The following theorem gives an error estimate for the full tensor approximation.

**Theorem 14** (Error estimate full tensor Galerkin method). *The full tensor Galerkin approximationu*_*L*,*N*_=P^*L*,*N*^*u* of a solution *u*∈*H*^*s*+1,0^(*Ω*)∩*H*^1,*t*^(*Ω*), *s*∈{0,1}, *t*∈{0,1}, *to the variational problem (21) satisfies the asymptotic error estimate*52

*with relation “ ≲” as in Lemma 7.*

*Proof*. By Céa’s Lemma (Brenner and Scott 2008, Thm. 2.8.1) the Galerkin approximation is quasi-optimal in *V*^*L*,*N*^, its error can therefore be bounded (up to constants) by the error of any other approximation to *u* in *V*^*L*,*N*^, for example the quasi-interpolated and *L*^2^-projected approximation : 

Here, we used the approximation properties of the quasi-interpolant from Lemma 9 and of the angular *L*^2^- projection from Lemma 12. The last step is a consequence of the *H*^1^-stability asserted in Lemma 10 of the quasi-interpolation. □

#### Error estimate for the sparse tensor phase space Galerkin method

After the full tensor approximation properties, we consider the convergence properties of a direct sparse tensor approximation on the sparse tensor product space  as defined in (45).

In analogy to the full tensor Galerkin projector P^*L*,*N*^, we can define a *sparse tensor Galerkin projector* by the orthogonality relation 

The error of the sparse tensor solution  is estimated in the following theorem (see also Widmer 2009, Thm. 2.6) and (Griebel and Harbrecht 2013a, Thms. 4.3 and 7.1)).

**Theorem 15** (Error estimate of direct sparse tensor solution). *Let the linear sparsity profile as in (44) be given. Assume further thatLandNvary such that* −*sL*+*tN*=*ζ*=*const, then the direct sparse tensor approximation**of a functionu*∈*H*^*s*+1,*t*^(*Ω*), *s*,*t*∈{0,1}, *satisfies the error estimate*

where relation “ ≲” is defined as in Lemma 7.

*Proof*. We follow the proof of Thm. 2.6 by (Widmer 2009). First we introduce so-called difference projectors  and  as the difference between projections to two consecutive resolution levels with the convention . They project onto the detail spaces  and , respectively.

With these difference projectors, a sparse quasi-interpolated and *L*^2^-projected approximation  to *u* can be expressed as 

 where  is the largest feasible angular resolution index which results from solving  with respect to .

Now we exploit quasi-optimality of the Galerkin approximation on the sparse tensor product space to replace the Galerkin approximation error by the error of the quasi-interpolated and *L*^2^-projected approximation. Additionally applying the norm estimate  yields 53

The error is split into two terms: 54

The second term on the right hand side can be estimated by Lemma 9: 55

This term will not contribute to the asymptotic terms.

The first term on the right hand side of (54) is split up further: 56

Both norms on the right hand side of (56) can be estimated by Lemma 9 and Lemma 12: 

Inserting back into (56) yields 57

The task is now to estimate the series. Using the assumption *ζ*=−*s*+*tN*/*L*: 58

We estimate the sum on the right hand side of (58) by its largest summand. Two cases can be distinguished here:

1. If *ζ*≤0, the largest summand occurs for *l*_*D*_=0: 

2. If *ζ*>0, the largest summand occurs for *l*_*D*_=*L*: 

In summary, we may write 

By combining this estimate with relations (53) to (57), we finally arrive at  □

In conclusion, we find that the convergence rate of *O*(2^−*sL*−*tN*^) of the full tensor approximation is maintained up to an additional factor *L*, which by *M*_*D*_=*O*(2^*dL*^) is logarithmic in the number of degrees of freedom. This result in conjunction with the greatly reduced complexity of the sparse tensor method (Lemma 7) shows its superior efficiency provided that the function *u* to be approximated is at least in *H*^*s*+1,*t*^(*Ω*), with *s*,*t*∈{0,1}.

## Numerical experiments

### Algorithms

For the numerical experiments we compute a sparse tensor solution with the help of the combination technique. The sparse solution is constructed according to the formula 

from a number of solutions  to the full tensor discrete variational formulation (38) of reduced physical resolution *ℓ*_*D*_ and angular resolution .

Clearly  is in the space , which is identical to the sparse tensor approximation space from (43). However, in general the combination approximation differs from a direct sparse approximation  (see also Grella 2013, Sec. 2.3.1). Due to the quasi-optimality of the direct sparse solution as an approximation in , the error of the combination approximation can serve as an upper bound (up to factors) for the error  of the direct sparse approximation.

Note that the convergence of the combination technique for the radiative transfer problem has not been shown formally yet. A recent proof for elliptic operators by (Griebel and Harbrecht 2013b) would be applicable under certain stability assumptions on the semidiscrete Galerkin projectors (for details we refer to (Grella 2013, Sec. 5.3.7)). However, the use of the combination technique approximation has practical advantages over the direct sparse approximation. First, to construct the subproblem solutions of lower resolution, an existing full tensor solver with standard nonhierarchical FEM bases can be reused, no direct sparse solver needs to be implemented. Second, the splitting into subproblems entails a natural level for parallelism in the algorithm, which can still be combined with parallel solution procedures at the level of each subproblem (an implementation is described in (Grella 2013, Chap. 7)).

Each of the full tensor subproblems is solved by a phase space Galerkin finite element method with nonhierarchical affine hat functions as physical basis and piecewise constants as angular basis. In the experiment of Sec. ‘Experiment 2’, the midpoint rule is used for angular quadrature which corresponds to the *S*_*N*_-method. However, in situations where ray effects (Lathrop 1968) pollute the results, adaptive quadrature may help (Stone 2007). As a simple adaptive rule we link the number of quadrature points *n*_*q*_ per dimension and per mesh element to the resolution levels *l*_*D*_,  of the subproblem by  in the experiment of Sec. ‘Experiment 1’. Even though the overall computational effort is then not bounded by Lemma 7, the total number of degrees of freedom still is. As the iterative, approximate solution of the linear system constitutes the most time consuming part, the sparse tensor method is, in practice, more efficient than the full tensor method.

### Quantities of interest

In applications, the radiative intensity is often coupled to other modes of energy transport via the net emission (e. g. Larsen et al. 2002, Eq. (1.1a)). The net emission can be computed in turn from the *incident radiation*59

For this reason, we choose the incident radiation as a lower-dimensional variable to visualize results and to analyze errors. The relative *L*^2^- or *H*^1^-error of the incident radiation is given by 

### Numerical experiments

All experiments are set on the domains *D*= [ 0,1]^*d*^, , with . We solve the RTP with isotropic scattering  and zero inflow boundary conditions *g*=0.

#### Experiment 1

We search the solution to the Gaussian blackbody radiation 

 with absorption and scattering coefficient *κ*=*σ*=1.

**Figure 1:**
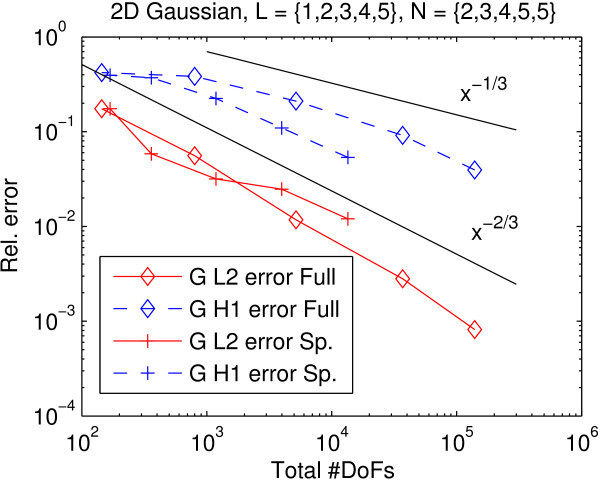
**Experiment 1: Convergence in incident radiation with full and sparse phase space Galerkin approximation.** Reference resolution was *L*_ref_=6/*N*_ref_=6. Reference slopes provided as visual aids only. Even with the lowest order sparse tensor phase space Galerkin discretization, the savings in DoFs to reach engineering accuracy of 1%–10% in the *H* ^1^ error is about an order of magnitude.

The *H*^1^-error of the incident radiation indeed converges faster in the sparse approximation than the full approximation (Figure 1). Note that the *L*^2^-error of the sparse approximation can be larger than the error of the full approximation because the sparsity profile  has been optimized for essentially undeteriorated convergence in the ∥·∥_1_-norm of the error in the radiative intensity, which is more closely represented by the *H*^1^-error than the *L*^2^-error of the incident radiation.

#### Experiment 2

A blackbody radiation *I*_*b*_(***x***,***s***) corresponding to the exact solution 

 with fixed  is inserted into the right hand side functional in (38) (Grella 2013, Sec. 8.2, Exp. 1). The absorption is set to *κ*=1, the scattering coefficient to *σ*=0.5.

For this experiment we employed a discrete ordinates solver in which the angular resolution *N*^′^ is related to the angular degrees of freedom by  so that *N*≈⌊log2(*N*^′^+1)⌋, where *N* is the angular resolution used otherwise in this paper.

**Figure 2:**
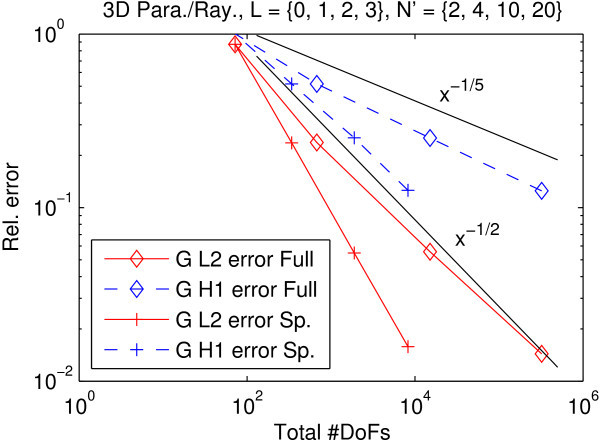
**Experiment 2: Convergence in incident radiation with full and sparse DOM.** Reference resolution was *L*_ref_=4. Angular resolution *N* ^′^ corresponds to *N*≈{1,2,3,4}. Reference slopes provided as visual aids only. The savings in DoFs to reach engineering accuracy of 1%–10% are about two orders of magnitude.

Figure 2 shows the superior efficiency of the sparse approach with respect to number of degrees of freedom vs. achieved error. The convergence rates indicate that the curse of dimensionality is mitigated by the sparse discrete ordinates method.

For a comparison to other sparse tensor approaches we refer to the numerical experiment section of (Grella 2013), which features a sparse tensor spherical harmonics approximation and a sparse collocation discrete ordinates method realized via the combination technique. We observed that the approach presented here performs similarly to the sparse collocation DOM combination technique as the methods are similar from the point of view of implementation, even though their theoretical derivation is different. The presented approach is somewhat less susceptible to ray effects at the expense of slightly longer computational times as the angular quadrature is adapted to the resolution of the angular mesh. The spherical harmonics method is most effective for solutions with highly regular angular part because of its regularity requirements for spectral convergence. In general, at the same resolution levels *L* and *N*, the combination technique approach realizes approximately the same error as the direct sparse approach, while the number of degrees of freedom in the combination technique is larger than in the direct sparse approach because the approximation spaces of different subproblems in the combination technique overlap in the degrees of freedom. It is therefore slightly less efficient than the direct sparse approach, but considerably more efficient than the full tensor approach and advantageous in practice due to faster and simpler implementation and parallelization.

## Conclusion

We have shown a direct sparse tensor phase space Galerkin approximation of the radiative intensity in the stationary monochromatic radiative transfer problem can be computed with only  degrees of freedom as opposed to  degrees of freedom for a standard full tensor approximation. Here, *M*_*D*_ is the number of physical degrees of freedom and  the number of angular degrees of freedom. At the same time, the error of the sparse approximation in the ∥·∥_1_-norm still decreases essentially as the error of the full approximation, namely with the order  as compared to  in the full tensor approximation. The parameters *s*,*t*∈{0,1} indicate the regularity of the exact solution which is required to be in the space of mixed smoothness  to achieve the sparse convergence rate, whereas  is sufficient in the full tensor approximation.

To simplify implementation, we realized the sparse tensor approximation algorithmically via the combination technique. Together with suitable quadrature rules, we demonstrated in numerical experiments that this sparse tensor combination approximation retains the analyzed theoretical advantages of the direct sparse tensor method while allowing for straightforward parallelization also at the level of subproblems.

The proposed specialization of the phase space Galerkin framework investigated here has the advantage that both discrete ordinates and spherical harmonics method can be derived from it so that the sparse tensorization benefits hold for the sparse variants of both methods alike.

Therefore, for problems whose solutions exhibit so-called mixed regularity, the sparse tensor product phase space Galerkin approximations realize a significant increase in efficiency, i. e. achievable error per number of degrees of freedom. Even in applications where high numerical accuracy is the main objective, a sparse tensor product approximation might be of value as an initial value for an iterative solver or in a problem-adapted preconditilpalitoccheme.
